# Dry eye disease and meibomian gland dysfunction among a clinical sample of type 2 diabetes patients in Ghana

**DOI:** 10.4314/ahs.v22i1.36

**Published:** 2022-03

**Authors:** Emmanuel K Abu, Amanfo O Ofori, Samuel B Boadi-Kusi, Stephen Ocansey, Richard K Yankah, Samuel Kyei, Asante Y Awuku

**Affiliations:** 1 Department of Optometry and Vision Science, School of Allied Health Sciences, University of Cape Coast, Ghana; 2 Diabetes Specialist Clinic, Cape Coast Teaching Hospital, Ghana; 3 School of Medical Sciences, College of Health and Allied Sciences, University of Cape Coast, Ghana

**Keywords:** Dry eye disease, meibomian gland dysfunction, type 2 diabetes, Ghana

## Abstract

**Objective:**

To evaluate dry eye disease and meibomian gland dysfunction among type 2 diabetes patients

**Methods:**

A hospital-based cross-sectional study was conducted. Parameters assessed included meibum expressibility and quality, Schirmer test 1, tear breakup time (TBUT), ocular surface staining, blink rates and Ocular Surface Disease Index (OSDI) scores. Dry eye was diagnosed based on a combination of subjective symptoms, tear function and ocular surface staining.

**Results:**

Prevalence of DED and MGD were 72.3% and 55.3% respectively. Symptomatic dry eye (OSDI scores) was significantly associated with duration of diabetes (rs = 0.11, P = 0.028) and the presence of conjunctival disorders (OR = 2.09, P = 0.002). MGD was a risk factor for DED (OR = 1.99, P = 0.008); ocular surface damage, the presence of eye lid lesions, abnormal Schirmer test and reduced TBUT were significantly associated with MGD, the strongest predictor being ocular surface damage (OR = 3.21, P = 0.001). OSDI scores had no association with the presence of corneal lesions possibly due to reduced corneal sensitivity.

**Conclusion:**

DED and MGD were prevalent among the patients and therefore there is the need for dry eye assessment as a routine clinical management protocol for patients with type 2 diabetes.

## Introduction

Dry eye disease (DED) was acknowledged by the International Dry Eye Workshop in 2007 as a lacrimal function unit (LFU) dysfunction.^1^,^2^ The lacrimal function unit regulates components of the tear film to maintain normal physiology of the ocular surface. It is composed of the cornea, conjunctiva, lacrimal gland, meibomian glands, eyelids, and the sensory and motor nerves that connect them.^3^ Due to their intricately close associations, systemic effects on any component of the LFU is easily transmitted to the entire structure via the neural connections, leading to tear function irregularities. The international dry eye workshop in 2017 defined DED as “a multifactorial disease of the ocular surface characterized by a loss of homeostasis of the tear film, and accompanied by ocular symptoms, in which tear film instability and hyperosmolarity, ocular surface inflammation and damage, and neurosensory abnormalities play etiological roles”.^4^ On the other hand, meibomian gland dysfunction (MGD) is “a chronic, diffuse abnormality of the meibomian glands, commonly characterised by terminal duct obstruction and/or qualitative/quantitative changes in the glandular secretion”.^5^ Even though the two are closely linked as MGD can alter the precorneal tear film composition and the fact that DED and MGD have similar symptoms, they are two separate disease entities.^6^,^7^

Diabetes mellitus (DM) is a known leading systemic risk factor for DED.^8^–^11^ Although the exact mechanisms accounting for dry eye in diabetes is not fully understood, some mechanistic pathways have been demonstrated. For instance, aldose reductase, an important enzyme of the polyol pathway, has been linked to the pathogenesis of dry eye in diabetes.^8^,^9^ The excess glucose in diabetes enters the polyol pathway where aldose reductase reduces the glucose to sorbitol whose retention within cells leads to cellular oedema.^8^ This results in lacrimal gland dysfunction and subsequently causing reduced tear secretion.^9^ Hyperglycaemia also causes damage to the corneal and conjunctival goblet cells thereby reducing mucin production and leading to tear film instability.^9^ Dysfunctions in the autonomic control of the lacrimal gland by diabetic neuropathy has also been associated with the suppression of hemodialysis-induced increases in tear film secretion.^10^ The risk of ocular manifestations in DM is linked to the duration of the disease and more importantly the degree of glycaemia control^11^,^12^ and therefore early diagnosis and prompt treatment are key to prevention of ocular complications. Ocular complications in diabetes pose serious public health concerns particularly in developing countries as a result of increased new cases and inadequate early tracking schedules.^13^

This study evaluated dry eye related parameters such as blink rate, Schirmer test, tear breakup time (TBUT), ocular surface epithelial damage, and the volume and quality of meibum expressed in type 2 diabetes patients reporting to a tertiary teaching hospital in Ghana. This study is the first report of dry eye investigation in patients with type 2 diabetes in Ghana. The results will improve the diagnostic protocol to guide and inform early and timely referral of diabetes patients for ophthalmic assessment including dry eye.

## Methods

### Study site

The study was conducted at the specialist diabetes clinics of the Cape Coast Teaching Hospital which is located in the Central Region, situated along the Atlantic Ocean. This teaching hospital serves as a referral centre for the Central, Western and some parts of the Eastern Regions of Ghana.

### Study Design and Participants

A hospital-based descriptive cross-sectional design was adopted in this study. A systematic sampling method was used to recruit confirmed type 2 diabetes patients reporting to the diabetes clinics of the Cape Coast Teaching Hospital. Type 2 diabetes was diagnosed according to the American Diabetes Association.^14^ Sample size was calculated using the formula N = Z^2^ (1 − p)(p)/b^2^,^15^ where N is the minimum sample size, Z is the standard normal deviation, usually set at 1.96 which corresponds to the 95% confidence interval, p is the proportion in the target population estimated to have the disease (6.7% for the Central Region),^16^ and b is the degree of desired accuracy, usually set at 5%. Subsequently, a sample size of 96 was calculated. This was adjusted to 300 participants to account for attrition rate and inefficiencies associated with the small sample size.

### Inclusion/exclusion criteria

Only confirmed type 2 diabetes patients were included. Subjects with type 1 diabetes, secondary diabetes and other conditions that could affect tear film dynamics were excluded. This included patients with the history of cigarette smoking, contact lens wear, LASIK surgery, allergies, rheumatoid arthritis, Parkinson's disease and systemic lupus erythromatosis. Again, all pregnant women and patients who were using medications such as antihistamines, tricyclic antidepressants, topical ophthalmic agents and oral contraceptives were excluded.

### Ethical considerations

The study was conducted in accordance with the tenets of the Declaration of Helsinki regarding research involving human subjects. Consent forms including local language versions which were issued or read out to each participant to sign or thumb-print were obtained prior to the commencement of the study. This was after the rationale and purpose of the study was explained to each participant in the language they understood best. The patients were guaranteed confidentiality and safety throughout the investigation. They were also informed that their participation was voluntary and that they could withdraw from the study at any given point. Ethical approval was obtained from the Institutional Review Board of the University of Cape Coast (UCCIRB/CHAS/2018/29). Permission to conduct this study was also obtained from the teaching hospital before the start of the study.

### Methods of data collection

Data collection methods comprised questionnaires, medical records, and ophthalmic examination. The questionnaires collected information on age, sex, occupation, and past general ocular screening. Patients' medical records were retrieved and their entire medical histories were reviewed to collect information on type of diabetes, duration of diabetes, associated systemic conditions, as well as current medications being used.

### Symptomatic dry eye assessment

Symptomatic dry eye disease was assessed using the OSDI questionnaires. It is a 12 item questionnaire that assesses the frequency at which patients experience dry eye symptoms such as painful or sore eyes, blurred vision, poor vision, gritty sensation, light sensitivity and how these symptoms limit them in daily activities like reading, watching television, driving at night, and working with a computer. Each of the 12 items is scored on a scale of 0 to 4: 0 designates none of the time, 1 indicates some of the time, 2, half of the time, 3, most of the time, and 4, all of the time. The total OSDI scores were then calculated based on the formula: OSDI = [(sum of scores for all answered questions) × 25] / (total number of answered questions). The OSDI questionnaire was thus scored on a scale of 0 -100, with higher scores indicating greater disabilities. Scores ranging from 0–12 were classified as normal, 13–22 as mild, 23–32, moderate and scores between 33 and 100 were classified as severe.^17^

### Clinical ophthalmic examination

All participants underwent anterior eye examination using the slit-lamp biomicroscope (Keeler, Halma UK) in a dimly illuminated room to ensure proper view of various structures with keen attention on eyelashes and lid margins, bulbar and palpebral conjunctiva, cornea, aqueous humor, iris, pupils and the crystalline lens. Clinical assessment included meibum expressibility and quality, Schirmer test 1, tear breakup time (TBUT), ocular surface staining, and blink rates. Clinical measurements were carried out for both the right and left eyes of each participant but the result of the worst eye was used in the analysis.

### Schirmer Test 1

A conventional Schirmer test 1 without anaesthesia, implying a measure of both basal and reflex tear secretion, was performed by placing the short portion of a folded Schirmer test strip (OptiTech, Eye Care Inc., India) at the outer one third of the temporal lower conjunctival fornix of both eyes and measuring the amount of wetting in mm at 5 minutes.^10^

### Tear breakup time

Fluorescein vital staining was used to determine TBUT. The tip of a moistened fluorescein strip (OptiTech Eye Care Inc, India), using a single drop of normal saline, was gently applied into the inferior cul-de-sac. After instillation, patients were requested to blink several times to ensure adequate mixing of fluorescein dye with tears. The time interval between the last complete blink and the appearance of the first random corneal dark spot under the slit lamp using the broad beam and cobalt blue filter was determined using a stopwatch as timer. The test was repeated three times and the mean of the three measurements was recorded in seconds as tear breakup time. Air conditions and fans in the examination room were switched off during this examination. A TBUT of less than 10s was considered abnormal in this study.^4^

### Ocular surface staining

The TBUT assessment was immediately followed by grading of ocular surface (cornea and conjunctiva) staining to evaluate the extent of ocular surface damage. Grading of ocular surface (cornea and conjunctiva) staining was done using the Oxford Grading Scale as described elsewhere^18^,^19^ where grade 0 means no staining; grade 1 indicates minimal staining; grade 2, mild staining; grade 3, moderate staining; grade 4, marked staining, and grade 5, severe staining. The test was also performed under cobalt blue filter. The patient was instructed to gaze nasally and temporally when grading the temporal and nasal zones respectively and also the upper lids were slightly elevated to grade the whole corneal surface.

### Blink rates

The blink rates of participants were assessed by letting them read letters off the logMAR visual acuity chart within a period of one minute. The number of blinks was noted by observation and this was done without the patients being consciously aware of the observations. A blink rate of less than 10 blinks per minute (bpm) was considered abnormal.^20^

### Diagnostic criteria

For the purposes of this research, dry eye was diagnosed based on the following three components: subjective symptoms (OSDI scores), tear function (TBUT) and vital staining test (Oxford grading scale).^21^ Subjects who were positive for at least two of the above three conditions were regarded as having dry eye disease. Subjects positive for only one condition or none at all were deemed non-dry eye patients.

### Assessment of meibomian gland function

A firm digital pressure was applied over the central and nasal one-third of the lower eyelid with the lid compressed against the globe until a dome of lipid was expressed from the orifices. Meibomian gland expressibility was assessed by focusing on the central 8 glands of the lower lid and observing the number of glands yielding lipid secretion under the slit-lamp biomicroscope. Gland expressibility was categorised as grade zero – all glands expressible (normal), grade one – three to four glands expressible, grade two – one to two glands expressible, and grade three – no glands expressible. The quality of expressed meibum was assessed by its clarity and viscosity, and graded as follows: zero – clear (normal), one – cloudy, two – cloudy with particles, and three – inspissated (toothpaste-like). The quality score was the highest grade for any of the expressed glands. Meibomian gland dysfunction (MGD) was diagnosed as: gland expressibility grade of one or greater and meibum quality score of one or greater in either eye.^22^

### Statistical analysis

The data was analysed using SPSS for Windows, version 21.0.1 (SPSS Inc., Chicago, IL, USA). Descriptive data was presented as percentages or as mean ± standard deviation or standard errors. Chi-square test was used to determine the associations between categorical variables. Independent-test and correlations were used for associations between the scores of dry eye clinical measures and risk factors, and linear and logistic regressions were used to determine predictive factors. A p - value ≤ 0.05 was considered statistically significant.

## Results

### Demographic characteristics

A total of 311 type 2 diabetes patients, 71 (22.8%) males and 240 (77.2%) females, aged 38 to 85 years with a mean age of 59.85 ± 10.043 years were examined. There was no significant difference between the mean ages of males (61.65 ± 11.03) and females (59.31 ± 9.69) (t = 1.73, P = 0.09). Duration of diabetes ranged from 1–35 years with a mean duration of 7.59 ±5.82 years. One hundred and twenty-one (38.9%) of the participants with a mean duration of 6.56±5.22 years had never undergone eye examination before.

### Clinical measures of dry eye disease

The mean OSDI score for all participants was 18.09±16.21 (indicating mild symptomatic dry eye), with scores ranging from 0 to 92.86. In terms of severity, OSDI scores were categorised as mild 60 (19.3%), moderate 33 (10.6%) and severe 62 (19.9%). Mean TBUT score was 7.03 ± 5.42 seconds with 213 (68.5%) having reduced values of less than 10 seconds. Schirmer values ranged from 1 to 35mm with a mean value of 15.45±0.10.95mm and a total of 167 (53.7%) participants getting reduced scores. Using the Oxford Grading Scale, 50 (16.1%) had Grade 0 (no staining), 60 (19.3%), Grade I (minimal staining), 90 (28.9%), Grade II (mild staining), 69 (22.2%), Grade III (moderate staining), 33 (10.6%), Grade IV (marked staining) and 9 (2.9%), Grade V (severe staining). Most (57.2%) participants had abnormal blink rates of less than 10 bpm, the mean blink rate being 8.98±6.963 and ranged from 1 to 41 bpm.

Two hundred and twenty five (72.3%) of the diabetes patients were diagnosed with DED. There was no significant difference in the prevalence of DED between males and females in this study (χ2 = 1.2, P = 0.27). One hundred and seventy two (55.3%) of the diabetes patients suffered from MGD. Meibomian gland dysfunction was not differently distributed among males and females (χ2 = 1.0, P = 0.31). Of the 172 diabetes patients who were diagnosed with MGD, 135 (78.5%) also suffered from DED. Conversely, out of the 225 patients who suffered from DED, 135(60%) had MGD. Thus, 135 (23%) of the patients were diagnosed with both DED and MGD at the same time.

### Associations between MGD/DED and clinical measures

Symptomatic dry eye (OSDI scores) positively correlated with duration of diabetes (rs = 0.11, P = 0.028) but no such association existed between MGD and duration of diabetes (rs = 0.09, P = 0.11). There were also no significant correlations between OSDI scores and any of the clinical measures of dry eye disease in the diabetes patients. However, MGD had significant associations with almost all clinical measures of DED. Thus, patients who had MGD were almost two times more likely to develop DED (OR = 1.99, CI: 1.20–3.29, P = 0.008). These MGD patients were also more likely to have ocular surface epithelial damage with the evidence of positive staining (OR = 3.16, CI: 1.66 – 6.01, P< 0.001). The relationship between MGD and clinical measures with counts were explored using correlation as shown in Table 1. TBUT values, Schirmer test scores, and blink rates, all had significant negative correlations with MGD scores. This meant that as these dry eye clinical test scores increased or improved, MGD values decreased or the meibomian glands became more functional. However, there was a positive correlation between age of patient and duration of diabetes and MGD scores, meaning that longer duration of diabetes and older patients suffered more MGD though the association was not significant regarding duration of the disease (Table 1).

**Table 1:** Correlation between MGD scores and clinical measures of DED

Clinical measure	rs	*P* - value
TBUT scores	− 0.20	.0.001*
Schirmer test scores	− 0.20	.0.001*
Blink rates	− 0.11	0.024*
Age of patient	0.15	0.009*
Duration of diabetes	0.09	0.11

* Correlation is significant at 0.05 level

*rs*, Correlation coefficient

All the clinical measures were included in a single logistic regression model to determine which clinical measures strongly predicted MGD in the diabetes patients. These factors were first analysed using a multiple linear regression model to identify which of them had a significant effect on the dependent variable (MGD) and to be included in the logistic regression model. Clinical tests: ocular surface staining, TBUT, and Schirmer tear test 1 had significant effects on the model, with ocular surface staining being the strongest predictor of MGD. Table 2 shows the relative positions of clinical tests predicting MGD.

**Table 2:** Relative positions of clinical tests predicting MGD

Clinical Test	Meibomian Gland Dysfunction		

	Relative Odds	95% CI	*P*-value
Presence of cornea staining	3.21	1.65 – 6.24	0.001
Schirmer (less than 15mm)	2.08	1.27 – 3.38	0.003
TBUT (less than 10s)	1.94	1.14 – 3.31	0.015

*CI*, Confidence interval

### Anterior segment abnormalities in the diabetes patients

Table 3 shows anterior segment abnormalities in the diabetes patients. The majority of anterior segment anomalies were conjunctival 302(97.7%), lid disorders 120 (38.6%), anomalies of eye lashes 69 (22.2%), cornea disorders 54 (17.4%) with iris abnormalities being the least 17 (4.5%). Patients who had eye lid lesions were two and half times more likely to develop MGD (OR = 2.53, CI: 1.51–4.24, P < 0.001) while the presence of conjunctival disorders was significantly associated with symptomatic DED (OR = 2.09, CI: 1.30–3.37, P = 0.002). The presence of cornea disorders was not, however, associated with either MGD or symptoms of dry eye (MGD: t = 1.26, P = 0.21; OSDI: t = 1.56, P = 0.12).

**Table 3:** Anterior segment abnormalities in the diabetes patients

Findings	n (%)
**Lashes**	**69 (22.2)**
Crusty/Frosty	32 (10.3)
Poliosis	22 (7.1)
Trichiasis	10 (3.2)
Madarosis	5 (1.6)

**Lids**	**120 (38.6)**
Thickening	62 (20)
Dermatochalazia	34 (10.9)
Notching	11 (3.5)
Warts	8 (2.6)
Stye/ Chalazia	4 (1.3)
Ptosis	1 (0.3)

**Conjunctiva**	**302 (97.1)**
Pterygium	111 (35.7)
Pigmented	89 (28.6)
Injected/Dilated tortuous vessels	59 (19)
Pinguecula	43 (13.8)

**Cornea**	**54 (17.4)**
Arcus	41 (13.2)
Panus	4 (1.3)
Neovascularisation	3 (1.0)
Strands/Straie	5 (1.6)
Keratic precipitates	1 (0.3)

**Iris**	**17 (4.5)**
Atrophy	12 (0.9)
Iridodialysis	2 (0.6)
Rubeosis iridis	1 (0.3)

### Associations between anterior segment disorders and duration of diabetes/ age of patients

As shown on table 4, patients who had longer duration of the disease were more likely to develop anterior segment abnormalities though the association was significant only in disorders of the lashes. Again, patients who had anterior segment abnormalities were significantly older than those who did not have (t = 3.2, P = 0.001).

**Table 4:** Associations between anterior segment disorders and duration of diabetes/ age of patients

Anterior Segment Disorders	Duration of DM Mean± SEM		t	*P*-value	Age of patient Mean ± SEM		t	*P*-value

	Present	Absent			Present	Absent		
Lids	7.59±0.41	7.58±0.56	0.01	0.989	63.69±1.02	58.15±0.066	4.63	<0.001*
Lashes	9.40±0.71	7.08±0.37	2.94	0.004*	62.65±1.26	59.06±0.63	2.63	0.009*
Cornea	8.65±0.64	7.27±0.38	1.77	0.078	64.53±1.06	58.44±0.64	4.66	0.000*
Lens	8.16±0.47	7.05±0.46	1.69	0.093	63.35±0.71	56.54±0.80	6.35	0.000*
Conjunctiva	7.66±0.52	7.55±0.43	0.16	0.876	60.77±0.67	58.11±1.04	2.24	0.026*
Combined Anterior Segment Disorders	7.72±.38	7.15±.64	0.73	0.464	60.84±0.63	56.59±1.21	3.2	0.001*

*DM*, diabetes mellitus; *SEM*, standard error of mean; *t*, independent sample t test

* Statistically significant

## Discussion

This clinical-based investigation is the first report on the assessment of DED and MGD in patients with type 2 diabetes in Ghana. Again, this report is based on a combination of symptoms and clinical tests. Asiedu et al found a prevalence of symptomatic dry eye of 44.3% among a healthy Ghanaian sample,^23^ which is lower than the present finding (72.3%). A recent publication also reported a lower prevalence of 21.7% among type 2 diabetes patients in Nigeria.^24^ Kamel et al^25^ in a case-control study among type 2 diabetes patients in Egypt indicated a prevalence of 70% which is comparable to the current study. Elsewhere in Spain, Johanna et al 26 have reported a prevalence of 76.3% among type 2 diabetes. Karki et al^27^ found 54.3% prevalence in a Nepalese diabetic population; same as prevalence of 54.3% in Iran.^12^

Hospital-based studies like the present one usually report higher prevalence rates than do population-based studies.^28^,^29^ Lack of consistency in dry eye diagnostic criteria has also made it difficult to compare findings across different studies. For example, the earlier study in Ghana 23 and the study in Nigeria^24^ used only symptomatic dry eye diagnosis based on the OSDI scores, while the study in Egypt 25 was based on only TBUT results. The study in Spain by Johanna et al^26^ used a combination of symptoms and clinical tests like the present study. Apart from differences in the diagnostic criteria, race and ethnicity have been consistently argued to be a predisposing factor in the aetiology of DED, with the Asian race being a risk factor.^30^ A recent study in the US among type 2 diabetes patients by Ward et al^31^ reported that Asian patients were most likely to have DED (OR= 1.49; P = 0.03) followed by White patients (OR: 1.19; P <0.001) while Black patients were least likely to have the disease (OR = 1.03; P < 0.99). The associated risk factor for the Asian race in DED has been related to anatomical differences in eyelid structure. Kim et al^32^ have documented increased eyelid tension in Asian populations and suggested that this may generate shear stress during blinking, leading to ocular surface epithelial damage and incomplete blinks that have been reported among the Asian race.

High prevalence rate of DED in this Ghanaian sample may be attributed to evaporative water loss from the eye due to conditions of low relative humidity, air pollution and exposure to high wind velocity.^30^ In spite of these limiting factors to comparison between studies, case-control studies have reported worse dry eye parameter scores in type 2 diabetes patients than in controls. 11,25 In patients with type 2 diabetes, all the three layers of the tear film seem to be affected by hyperglycaemia leading to both aqueous tear-deficient and evaporative dry eye disease. This was particularly true in the current study as all dry eye related parameters (Schirmer test 1, TBUT, corneal staining, and blink rates) were lower than in a healthy Ghanaian sample.^20^ Reduced aqueous production as a result of lacrimal gland dysfunction has been associated with the action of increased aldose reductase in the sorbitol pathway.^8^ It has been established that oral administration of aldose reductase inhibitors could significantly improve tear dynamics.^33^ In the normal population, DED is known to be significantly frequent in females as a result of hormonal changes.^34^ The present study found no differences in the distribution of DED between males and females. Other studies have also reported no female preference for dry eye and have suggested that existing associations of females to dry eye are neutralised in patients with diabetes.^27^,^24^,^35^ It is, however, not clear how the hormonal activity becomes neutralised.

We also found a higher prevalence of MGD of 55.3% compared to 25.5% among a Ghanaian healthy youthful sample with a lower mean age of 21.8 years.^36^ From Spain, Johanna et al^26^ reported the prevalence of MDG of 75.6% among patients with diabetes and 66.67% in controls. A study in India also found 15% prevalence in diabetics and 7% for the non-diabetic controls.^37^ The wide variation between the findings of Johanna et al and that of the Indian study may be due to the wide age differences among the two study participants. Osae et al^38^ reported that 84% of dry eye patents in Ghana were diagnosed with MGD; that finding was higher than the present study where 60% of the dry eye patients were also diagnosed with MGD while 78.5% of the patients with MGD suffered DED as well. Thus, while DM is a major risk factor for both MGD and DED, MGD in turn presents as a further risk factor for DED. Arita et al^7^ found among a Japanese population that 38.7% of dry eye patients were diagnosed with MGD with only 12.9% of their study sample having both DED and MGD. The present study, however, found 23% of our sample having coexistence of both conditions where patients who had MGD were two times more likely to develop DED as also reported by Johanna et al^26^ of OSDI scores being significantly higher in the diabetic group.

Our study also revealed that MGD patients were three times more likely to have epithelial damage as revealed by the staining procedure. The implication is that abnormal meibum quality and quantity leading to reduced tear film lipid layer, tear hyperosmolarity and the onset of inflammatory cascades,^6^ the ultimate sequel is damage to the ocular surface. Johanna et al^26^ and Shamsheer et al^35^ have also reported significantly increased corneal staining in a diabetic group as compared to the non-diabetic controls. Corneal complications in DM has also been associated with epithelial barrier dysfunction which leads to the destruction of conjunctiva and cornea goblet cells, reducing mucin production and resulting in tear film instability.^9^ Dogru et al^11^ confirmed this when they established significantly reduced goblet cell density in patients with diabetes as compared to controls. Meibomian gland dysfunction is a major cause of evaporative dry eye which results in reduced TBUT values^1^ as recorded in this study; not only were TBUT values reduced but they negatively correlated with MGD scores, indicating that MGD resulted in reduction of tear quality. Compromise in tear quality in DM has been linked to hyperglycaemic related destruction of the meibomian glands leading to deficient meibum volume and quality.^1^,^24^ Aqueous tear deficiency evidenced by reduced Schimer test scores in the present study could explain hyperglycaemic related neuropathic impairment of lacrimal gland innervation in DM, reducing aqueous tear secretion.^4^ Hyperglycaemic associated cornea neuropathy also results in reduced corneal sensitivity, which decreases blink rate.^1^ It was therefore not surprising that we found no significant associations between the presence of cornea lesions and dry eye symptoms. This could be as a result of reduced sensitivity of the cornea to the presence of the disorders. The presence of eye lid and conjunctival disorders were, however, associated with MGD and dry eye symptom scores respectively. Several anterior segment disorders were found in this study, similar to a previous study.^39^ These ocular complications in DM are linked to the duration of disease^11^,^12^ similar to our findings.

The high prevalence of DED and MGD in the diabetes patients has clinical implications for practitioners. Since DED has been found to induce psychological conditions such as depression and anxiety,^40^ persons diagnosed with diabetes need prompt referral for dry eye assessment and subsequent treatment so that these patients do not develop worse general health conditions. Prompt treatment has been associated with improvements in OSDI scores, disease-specific quality of life and patients' ability to perform activities of daily living. 41 The fact that almost 40% of the patients had never undergone any previous ophthalmic assessment reveals the lack of access and/or failure of a referral protocol by practitioners. This calls for a standard referral protocol for all diabetes patients to undergo ophthalmic assessment including dry eye. Several treatment options exist for DED in such high risk populations like diabetes patients. These include counselling in relation to environmental modifications and lifestyle changes such as controlling humidity, dietary changes, cessation of smoking, eyelid hygiene, avoidance of certain medications, and the use of pharmacological agents. 4,41

## Limitation

The results in the patients were not compared with control subjects but rather with previous studies and therefore interpretation of the results should be done with caution. Another limitation is that many of the patients with type 2 diabetes also had systemic hypertension and were receiving antihypertensive medications. Ocular side effects of some antihypertensive drugs include dry eye and its related conditions.^42^ It is therefore possible that the use of these medications could contribute to the high prevalence of DED and MGD in the present study. These limitations notwithstanding, this study reports for the first time the prevalence of DED and MGD among patients with diabetes in Ghana and that ocular surface epithelial damage is the strongest predictor of MGD in these patients.

## Conclusion

DED and MGD were found to be highly prevalent among type 2 diabetes patients and thus elucidates the importance of regular referral for ophthalmic assessment including dry eye as a routine clinical management protocol of individuals diagnosed with type 2 DM.
